# The NF-κB Pathway and Cancer Stem Cells

**DOI:** 10.3390/cells5020016

**Published:** 2016-04-06

**Authors:** Amanda L. Rinkenbaugh, Albert S. Baldwin

**Affiliations:** 1 Department of Pathology and Laboratory Medicine, University of North Carolina, Chapel Hill, NC 27599, USA; arink@email.unc.edu; 2 Lineberger Comprehensive Cancer Center, University of North Carolina, Chapel Hill, NC 27599, USA

**Keywords:** NF-κB, cancer, cancer stem cells, tumor-initiating cells, self-renewal

## Abstract

The NF-κB transcription factor pathway is a crucial regulator of inflammation and immune responses. Additionally, aberrant NF-κB signaling has been identified in many types of cancer. Downstream of key oncogenic pathways, such as RAS, BCR-ABL, and Her2, NF-κB regulates transcription of target genes that promote cell survival and proliferation, inhibit apoptosis, and mediate invasion and metastasis. The cancer stem cell model posits that a subset of tumor cells (cancer stem cells) drive tumor initiation, exhibit resistance to treatment, and promote recurrence and metastasis. This review examines the evidence for a role for NF-κB signaling in cancer stem cell biology.

## 1. Introduction to NF-κB Signaling

**Figure 1:**
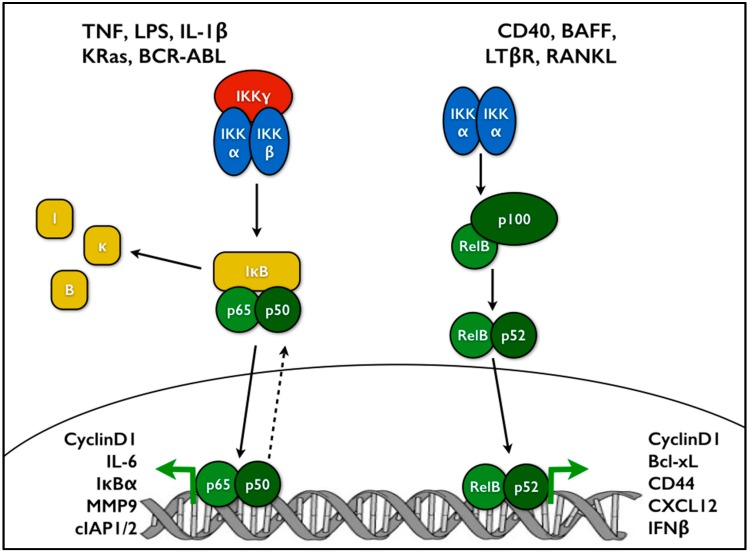
NF-κB signaling consists of two main branches: canonical and non-canonical. On the left, canonical NF-κB is driven by the IKK complex containing IKKα, β, and γ subunits. Phosphorylation of IκBα leads to its degradation, allowing the p65-p50 dimer to accumulate in the nucleus and regulate transcription of target genes. On the right, non-canonical NF-κB is driven by IKKα homodimers, leading to p100 processing. Here the dimer consists of the RelB and p52 subunits. Canonical and non-canonical NF-κB subunits regulate expression of distinct and overlapping sets of target genes [7].

The NF-κB family of transcription factors consists of five members: p65 (RelA), RelB, c-Rel, p105/p50, and p100/p52. Each of these proteins contains a conserved N-terminal Rel homology domain which enables nuclear localization, DNA binding, and homo- and heterodimerization. p65, RelB, and c-Rel feature transcription activation domains as well, while p50 and p52 do not. The precursors p105 and p100 include ankyrin repeats which are proteolytically cleaved to produce the active subunits p50 and p52, respectively [1]. NF-κB signaling typically operates through two major pathways: the canonical and the non-canonical. Activation of the canonical pathway occurs downstream of many stimuli, including LPS and pro-inflammatory cytokines such as TNF or IL-1. Under basal conditions, p65-p50 dimers are bound by the inhibitor of κB proteins (IκBs), which shift the steady state localization of NF-κB to the cytosol, while still allowing nucleocytoplasmic shuttling [2,3,4,5]. Upon activation, the inhibitor of κB kinase (IKK) complex, which consists of the kinase subunits IKKα and IKKβ plus the regulatory subunit IKKγ (NEMO), phosphorylates IκBα, leading to its ubiquitination and proteasomal degradation. Loss of IκBα enhances NF-κB nuclear accumulation and DNA binding, promoting transcription of its target genes, including anti-apoptotic factors, cytokines such as IL-6, and proliferation factors such as cyclin D1 (Figure 1). One group of target genes includes negative regulators of NF-κB signaling, such as A20 and IκBα. By producing these components, NF-κB generates a negative feedback loop to add another dimension of control to this pathway. The non-canonical pathway is activated through developmental signals such as BAFFR, CD40, or LTβR. Here, p100 acts like an IκB molecule, holding RelB in the cytoplasm. Non-canonical signaling leads to stabilization of NF-κB-inducing kinase (NIK). NIK activates IKKα dimers, which subsequently phosphorylate p100. p100 phosphorylation leads to its cleavage into p52, producing an active RelB-p52 dimer that moves to the nucleus and regulates transcription (Figure 1) [6].

Given that NF-κB signaling is tightly intertwined with cytokine production and immune responses, it was natural to investigate a role for NF-κB in hematopoietic cells. Germline knockout of RelB resulted in mice with myeloid hyperplasia and extramedullary hematopoiesis [8]. Double knockout of cRel and RelA produced cells with impaired engraftment and erythropoiesis, along with deregulated granulocyte expansion [9], suggesting distinct, yet overlapping roles for different NF-κB subunits. Subsequent studies built on these findings to suggest that the level of NF-κB activation was tightly regulated in hematopoietic stem cells (HSCs). RelA deletion in HSCs leads to changes in gene expression consistent with decreased HSC maintenance and homeostasis with a concomitant increase in genes associated with lineage restricted cells [10]. Interestingly, non-canonical NF-κB was also found to contribute to HSC self-renewal, both intrinsically and through microenvironment interactions [11]. Others have found that activation of NF-κB, either through TLR activation or loss of miR-146, actually enhances myeloid differentiation of HSCs [12,13]. A similar paradigm is observed in embryonic stem cells, which are reported to have a low level of NF-κB activation. Nonetheless, inhibition of NF-κB drives differentiation of these cells [14,15]. Conversely, overexpression of p65 enhanced differentiation and loss of pluripotency in embryonic stem cells [16], consistent with the need for balanced NF-κB activity. Results in muscle stem cells show decreased canonical NF-κB signaling during differentiation, but a late induction of non-canonical NF-κB, demonstrating that these pathways can have distinct roles in stem cell biology [17,18,19]. Taken together, these studies illustrate the involvement of NF-κB signaling in the maintenance of a variety of stem cells, consistent with much of the literature on NF-κB in cancer stem cells.

## 2. NF-κB in Cancer

### 2.1. NF-κB Activation in Cancer

In addition to its roles in the innate immune system and inflammatory signaling, the NF-κB pathway has been extensively tied to cancer biology. The discovery of v-rel, the oncoprotein in an avian Rev-T virus responsible for reticuloendotheliosis, and its identification as the homolog of c-rel provided the first link between cancer and NF-κB [20,21]. Early efforts showed that NF-κB is activated downstream of oncogenic RAS and BCR-ABL, where it promotes the oncogenic phenotype [22,23,24]. Inhibition of NF-κB in oncogenic RAS+ cells leads to apoptosis, consistent with a role for NF-κB in driving an anti-apoptotic, pro-survival phenotype [25]. Many studies demonstrate that NF-κB and its target genes are upregulated in the majority of cancers—including both hematological malignancies and solid tumors. More recently, NF-κB has been shown to be activated downstream of loss of tumor suppressors such as p53, VHL, and PTEN [26,27,28,29,30,31,32,33,34]. While early efforts focused on analysis of canonical NF-κB signaling in cancer, recent studies indicate that non-canonical NF-κB signaling can also be found activated in different cancers [35,36,37,38,39,40,41]. Expression of the superrepressor form of IκBα (serines 32/36 mutated to alanines, preventing phosphorylation and degradation and leading to decreased NF-κB activity; IκBα-SR) and genetic deletion of IKKβ or RelA in RAS-driven lung tumor and melanoma models strongly suppressed tumor growth [42,43,44].

Once activated, NF-κB regulates a wide variety of target genes that overlap heavily with the hallmarks of cancer [45]. Proliferation is one of the most basic characteristics of a cancer cell and NF-κB is involved through regulation of CyclinD1, Cyclin E, and c-Myc. NF-κB promotes survival and inhibits apoptosis through several mechanisms [46]. These include transcriptional regulation of the cellular inhibitor of apoptosis (cIAPs) 1, 2 and XIAP, as well as Bcl-2 and Bcl-xL [47,48,49]. Perhaps, as expected, NF-κB regulates a number of cytokines that contribute to tumor-promoting inflammation such as: TNFα, IL-1, IL6, MCP1, COX2, and iNOS. Other NF-κB targets contribute to epithelial-mesenchymal transition (vimentin, Twist), remodeling the extracellular matrix through induction of angiogenesis (IL8, VEGF), and promotion of invasion and metastasis (MMP2, MMP9, uPA) [50].

The studies described above led to efforts to determine if human tumors feature genetic alterations in IKK/NF-κB components. Somewhat surprisingly, such mutations are not common. However, the level of coverage provided by next-generation sequencing has found examples of NF-κB-associated mutations in a low percentage of cancers, predominately hematological malignancies. Amplifications of c-rel, IKKβ, IKKγ, and the related kinase IKKε have been identified primarily in lymphomas and breast cancer [51,52,53]. Rearrangements of the NFKB2 locus (gene name for the p100 subunit) that lead to loss of the inhibitory IκB-like domain and increased p52 production are found in some B cell lymphomas [54]. C11orf95-RELA fusions have been described as driver events in ependymomas [55], while monoallelic deletions of IκBα were identified in a subset of glioblastoma [56]. Mutations in upstream proteins that lead to aberrant, constitutive NF-κB activation have been identified. For example, in certain subtypes of lymphoma, translocations can affect MALT1 and BCL10, while CARD11 features a variety of point mutations. All three of these proteins interact to form a complex that drives NF-κB activation [57]. Growth factor receptors, including EGFR and Her2, are frequently overexpressed in cancer and activate similar pathways, including NF-κB [58,59].

IKK exhibits NF-κB-independent functions that promote growth and survival functions important to a variety of cancer cells. For example, IKKα and IKKβ promote mTOR activation, via kinase activity [60,61,62,63]. Another example is that IKKα was found to phosphorylate the CDK inhibitor p27 downstream of Her2 to promote cancer stem cell self-renewal [64]. IKKβ was reported to phosphorylate the tumor suppressor p53 to promote its instability [65].

### 2.2. Chronic Inflammation as a Precursor to Cancer

Another line of research linking NF-κB with oncogenesis examines the connection between chronic inflammation and tumor development. The microenvironment surrounding a tumor includes fibroblasts, infiltrating immune cells, extracellular matrix proteins, and cytokines that interact with the tumor cells. In a mouse model of colitis-associated colon cancer with targeted IKKβ deletion in either the epithelial or myeloid compartments, NF-κB mediated survival of intestinal epithelial cells, while NF-κB activation in myeloid cells drove production of growth factors that promoted tumor proliferation [66]. In a model of hepatocellular carcinoma driven by treatment with the carcinogen diethylnitrosamine (DEN), NF-κB is again activated in the myeloid compartment, this time to drive IL6 production and subsequent STAT3 activation in the hepatocytes [67]. Interestingly, this model shows that deletion of IKKβ or IKKγ actually leads to enhanced tumor development. The liver initially shows more cell death following DEN treatment, however, because hepatocytes are highly regenerative, the cell death triggers proliferation of the remaining cells [68]. It is thought that a cycle of injury, cell death, and proliferation drives tumor formation in this model [69]. NF-κB was shown to be activated in cancer-associated fibroblasts promoting the expression of inflammatory cytokines, although the role of this response in promoting tumorigenesis is controversial [70,71,72]. Work examining tumor-associated macrophages has shown that NF-κB signaling maintains a tumor-promoting, immunosuppressive (or M2) phenotype and inhibits a tumor-suppressing (or M1) phenotype [73,74]. Taken together, these studies start to describe a complex microenvironment with multiple cell types interacting to drive tumorigenesis and place NF-κB as a central mediator between these various components.

## 3. Cancer Stem Cells

Given the connections between the NF-κB pathway and the earliest events in oncogenesis, it follows that NF-κB signaling would be important in the tumor initiating cells. The cancer stem cell (CSC) model has been proposed to describe the cells which are responsible for tumor initiation. This phenomenon was first described in acute myeloid leukemia (AML), where cells from patients were transplanted into NOD/SCID mice and monitored for engraftment. Results from that study demonstrated that the CD34^+^ CD38^−^ population of cells caused disease more frequently and at lower cell numbers than CD34^−^ cells [75]. Subsequent to these findings, CSCs have been described in many solid tumors including those of the brain, prostate, breast, colon, and pancreas [76,77,78,79,80,81]. In addition to being responsible for primary tumor formation, CSCs are also generally thought to drive metastasis and exhibit increased resistance to radiation and chemotherapy. Due to their stem-like characteristics, these cells are also capable of differentiation into multiple lineages, which accounts for some of the heterogeneity seen in tumors [82]. While CSCs are frequently depicted at the top of a hierarchically arranged tumor, there is evidence that plasticity allows for the conversion of bulk tumor cells into CSCs [83].

Several assays allow for the study of CSCs. *In vitro* experiments focus on sphere formation under stem cell permissive conditions, such as serum-free media supplemented with essential growth factors and low-adherence plates. Ideally, these experiments are performed at limiting dilutions to best assess self-renewal from single cells. Additionally, *in vivo* assessments of tumor formation remain the gold standard for true CSC function, again preferably performed under limiting dilutions [84,85,86,87]. Frequently, prospective CSCs can be isolated from the bulk of the tumor cells based on one or more markers, either through the use of magnetic beads or fluorescence-activated cell sorting. Many markers have been proposed to distinguish CSCs from other tumor cells. While no individual marker is perfect, a few of the most commonly used markers include CD133, CD44 and EpCAM [76,78,79,81,88,89]. Once isolated, the populations can be compared in a number of phenotypic assays to dissect the differences between the cell types. Proliferation, survival, and gene expression analyses are commonly measured.

### 3.1. NF-κB Activation in CSCs

One of the earliest examples of NF-κB involvement in CSCs came from primary AML samples, where the CD34^+^ cells showed enhanced NF-κB DNA binding that was not seen in regular hematopoietic stem cells [90]. Since that initial report, elevated or constitutive NF-κB activity has been seen in many tumor types. Prostate CSCs were found to express higher levels of acetylated and total p65, as well as a decrease in IκBα expression when compared to parental tumors [91]. In glioblastoma, CSCs exhibited increased nuclear localization of p65 as compared with cells cultured under monolayer conditions [92]. Tumorsphere-forming cells showed increased phosphorylation of p65, again consistent with elevated NF-κB signaling in this population of cells. In that study, inhibition of NF-κB reduced self-renewal and blocked xenograft tumor growth using a limiting dilution approach [93]. In addition to direct evidence of preferential NF-κB activation in CSC subsets of tumors, several groups have taken an unbiased approach of profiling gene expression and defining CSC-associated signatures. This has revealed an inflammatory signature, which can frequently be tightly associated with NF-κB regulation, in a variety of tumors such as glioblastoma, breast, prostate, and ovarian cancers [94,95,96,97,98,99].

**Figure 2:**
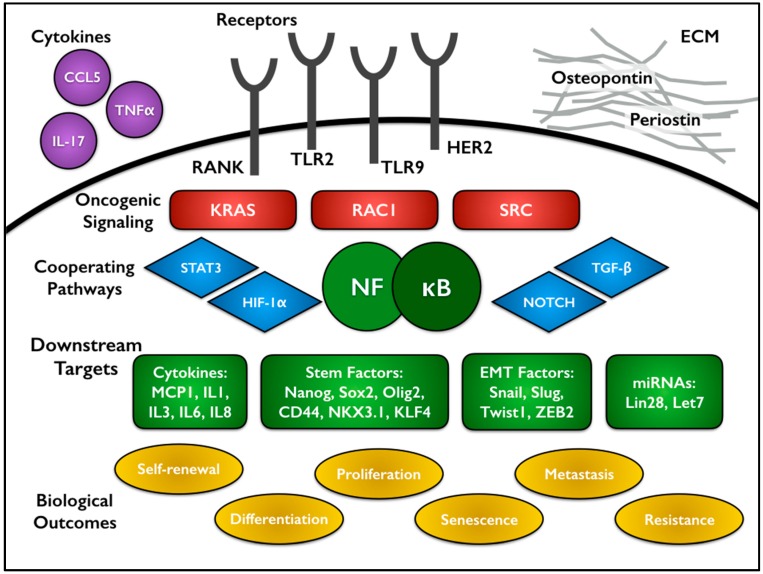
This diagram summarizes the various levels of NF-κB signaling in cancer stem cells. Both extracellular and intracellular sources of NF-κB activation are seen at the top. Either alone or in cooperation with other signaling pathways, NF-κB mediates a wide variety of transcriptional targets, which fall into several major categories such as cytokines and EMT factors. Ultimately, these targets mediate important aspects of CSC biology, including self-renewal, proliferation, and metastasis.

Perhaps not surprisingly, some of the same oncoproteins previously mentioned to activate NF-κB also participate in the CSC subpopulations of tumors. In mouse models of Her2-driven breast cancer, both canonical and non-canonical NF-κB pathways contribute to stemness and tumor formation. Expression of IκBα-SR impaired the formation of luminal epithelial tumors. Use of an NF-κB-GFP reporter allele localized activation to the luminal progenitors [100]. Another analysis of IκBα-SR in a Her2 mouse model found changes in a gene signature associated with stem cells, then specifically showed NF-κB-dependent changes in the specific stem cell factors Nanog and Sox2 (Figure 2) [101]. Knock-in of a kinase dead IKKα led to decreased self-renewal and senescence under mammary stem cell culture conditions [102]. In the Her2 breast cancer model, IKKα was found to phosphorylate p27 leading to its nuclear export and promoting CSC proliferation and expansion [64]. One of the canonical alterations that occurs during colorectal tumorigenesis is loss of APC. Myant and colleagues found that APC loss drives RAC1 activity to mediate ROS production and NF-κB activation, ultimately leading to an expansion of Lgr5^+^ CSCs [103].

### 3.2. Connections between NF-κB Signaling, Cytokines, and CSCs

Signaling from toll-like receptors (TLRs) is known to drive traditional NF-κB activation in an inflammatory setting. In ovarian CSCs, TLR2-MyD88-driven NF-κB activity regulates expression of the stem cell associated genes CD44, Sox2 and Nanog [104]. TLR9 drives the propagation and self-renewal of androgen-independent prostate CSCs, largely through the co-activation of the NF-κB and STAT3 pathways, which in turn regulate expression of the crucial stem cell transcription factors NKX3.1 and KLF4 [105]. Numerous cytokines have also been associated with supporting CSC maintenance in an NF-κB-dependent manner. Chronic myeloid leukemia (CML) stem cells produce higher levels of TNFα than normal hematopoietic stem cells. Canonical NF-κB activation positively regulates expression of IL-3 and granulocyte/macrophage colony-stimulating factor common β-chain receptor (CSF2RB) to promote proliferation and survival of CML stem cells [106]. Similar findings in a mouse model of acute myeloid leukemia (AML) described a feedback loop between TNFα and NF-κB, confirmed by correlations in patient samples [107]. TNFα treatment of MCF7 breast cancer cells increased their mammosphere-forming capacity through upregulation of NF-κB and subsequently Slug (Figure 2) [108]. In colorectal cancer, levels of prostaglandin E_2_ (PGE_2_) correlated with CSC markers in human tumor samples. Treatment of either a genetic or xenograft mouse model with PGE_2_ led to CSC expansion through upregulation of several signaling pathways including NF-κB [109]. In glioblastoma, IL-17 receptor was found to be co-expressed with multiple CSC markers, including CD133, Nestin, and Sox2, as well as a source of NF-κB activation [110].

While several cytokines drive NF-κB signaling, NF-κB also controls the expression of a variety of other cytokines, particularly IL-6 and IL-8, which are heavily associated with CSC function. Iliopoulous and colleagues studied an inducible model of transformation by Src in mammary epithelial cells that led to rapid secretion of IL-6 and increased NF-κB activation. NF-κB positively regulates Lin28 transcription, which in turn decreases the level of let-7 microRNA. As IL-6 is one target of this microRNA, IL-6 expression increases even further, creating a positive feedback loop driving transformation and CSC expansion [111,112]. Interestingly, let-7 also targets KRas, and decreased levels of let-7 have been shown to drive mammosphere formation and size through Ras-NF-κB and Ras-MAPK-ERK pathways [113]. In basal-like breast cancer, NF-κB inhibition decreases mammosphere formation, but addition of exogenous IL-6 or IL-1β rescues the defect [93]. In CML, increased levels of IL-6 drive CML progenitors into the myeloid lineage, sustaining CML development [114]. IL-6, IL-8, and MCP1 similarly contribute to the survival and self-renewal of glioblastoma CSCs (Figure 2) [110,115].

### 3.3. Interactions between NF-κB and the Tumor Microenvironment

Given the close association between NF-κB and cytokines, it reasonably follows that NF-κB plays a role in modulating the microenvironment. CSCs are thought to occupy certain niches within tumors, much like their normal stem cell counterparts. For example in glioblastoma, CSCs have been localized to a perivascular niche, populated by an abundance of proliferating stromal and endothelial cells that support the growth of CSCs specifically [116,117]. As previously mentioned, preferential expression of IL-17 receptor is seen on glioblastoma CSCs. Relatedly, in ovarian cancer, macrophages and CD4^+^ T cells produce IL-17 to drive self-renewal of CSCs *in vitro* and tumor formation *in vivo* in an NF-κB- and p38-dependent manner [118]. Interestingly, there is evidence for CSCs promoting angiogenesis through secretion of endothelial factors like VEGF and IL-8 or through direct transdifferentiation [80,119,120,121]. Osteopontin is an oncoprotein that signals through integrins as well as CD44 family receptors, which have been used as a CSC marker in several tumor types. Hepatocellular carcinoma stem cells exhibit enhanced expression of osteopontin which drives a transcriptional cascade from NF-κB activation to HIF1α to BMI1 expression [122]. Periostin (POSTN) is a component of the extracellular matrix that has been identified in the niche of both normal and cancer stem cells. Generally thought to be produced by stromal fibroblasts, POSTN promotes metastasis to the lung in a breast cancer model by supporting the growth and expansion of CSCs [123]. Another group found that *in vitro* breast CSCs express higher levels of POSTN than their non-CSC counterparts. POSTN drives an ERK-NF-κB signaling axis, driving production of IL-6 and IL-8, which in turn contribute to CSC maintenance through STAT3 activation [124]. Breast cancer also exhibits a circuit of progestin-driven RANKL (receptor activator of NF-κB ligand) expression, leading to NF-κB activation. Deletion of the RANKL receptor RANK decreases the CD49f^hi^-CSC population and tumor incidence (Figure 2) [125].

### 3.4. Contributions by the NF-κB Pathway to Invasion and Metastasis

In addition to creating the proper niche for CSC survival and expansion, NF-κB also contributes to the invasive and metastatic capabilities of CSCs. This can occur through further modulation of the extracellular environment or through cell-intrinsic changes like epithelial-mesenchymal transition (EMT) which has been linked to CSC characteristics [126]. Work by several groups has shown NF-κB-mediated regulation of critical EMT factors including Snail [127,128], Slug [129,130], ZEB1/2 [131], and Twist1 [132,133,134,135,136] (reviewed in [137]). TNFα leads to NF-κB-dependent stabilization of Snail and transcriptional upregulation of Twist1, both of which enhanced invasion *in vitro* and metastasis *in vivo* [138,139]. Inhibition of NF-κB led to a reversal of EMT in mammary epithelial cells and decreased metastasis in an *in vivo* model [140]. In breast cancer, overexpression of RANK drives EMT and expansion of the CD44^+^/CD24^−^ CSC population, ultimately leading to increased tumor growth and a substantially higher number of metastases [141]. Another study found overexpression of AXL in breast cancer stem cells; inhibition of AXL decreased NF-κB activity, expression of EMT-associated genes, invasion, and tumor formation [142]. In non-small cell lung cancer (NSCLC), Kumar and colleagues induced EMT through dual treatment with TNFα and TGF-β. The associated EMT transcription factors Twist1, Slug, and ZEB2 were upregulated in an NF-κB-dependent manner, followed by increases in multiple stem cell factors: KLF4, SOX2, POU5F1, MYCN, and KIT [130]. Subsequent studies found that NF-κB-mediated upregulation of Activin was required in order to maintain the mesenchymal phenotype of NSCLC CSCs [143]. There is also evidence that signaling through the NF-κB pathway and CXCR4 maintains stemness and promotes migration [144,145,146,147]. NF-κB has also been found to regulate the expression of matrix metalloproteinases (MMPs), which can degrade components of the extracellular matrix to increase invasion of tumor cells. Specifically, NF-κB directly regulates transcription of MMP9 [148,149,150], while indirectly increasing MMP2 activity [151,152,153]. Ovarian CSCs upregulate MMP9 expression to enable invasion and metastasis downstream of CCL5-NF-κB signaling [154]. NF-κB has also been shown to regulate VEGF and IL-8, which promote tumor formation and angiogenesis [155].

NF-κB frequently cooperates with additional signaling pathways to mediate these oncogenic effects. Coordinated activity between NF-κB and STAT3 has been previously mentioned in this review. Concurrent constitutive signaling from NF-κB and STAT3 in glioblastoma CSCs regulates expression of a set of genes (NOTCH1, HES5, JAG1, NUMBL, DTX3, DVL3, and RBPJ) that drive activation of Notch signaling, a third CSC-associated pathway (Figure 2) [92]. Another experiment, suggesting an important interaction between the CSCs and the bulk tumor cells, found that NF-κB activity in the non-CSCs upregulates JAG1 to stimulate Notch signaling in proximal breast CSCs [156].

The majority of the findings discussed here have focused on the canonical NF-κB pathway, particularly the p65 subunit. However, there is also evidence that the non-canonical pathway contributes to CSC phenotypes. In breast cancer, knockdown of IKKα, p100/p52, or RelB all produced a decrease in mammosphere formation [93]. Eva1, found to be overexpressed in glioblastoma CSCs, drives NIK stabilization and p100 processing, potentially by promoting ubiquitination and degradation of TRAF2 and cIAP [157]. RelB has been described as an oncogenic driver in mesenchymal glioma, regulating Olig2 expression and promoting tumor growth and invasion [39].

## 4. NF-κB as a Therapeutic Target

Given the extensive ties between NF-κB signaling and cancer biology, there has naturally been an interest in targeting the pathway therapeutically. In several of the studies previously mentioned, either knockdown of pathway components or targeted inhibitors produced a decrease in stem cell phenotypes *in vitro* as well as decreased tumor growth and/or metastasis *in vivo*. A combination of idarubicin and MG132, a proteasome inhibitor, induced cell death in AML stem cells, partially through NF-κB inhibition [158]. While proteasome inhibition will impact several pathways in a cell, NF-κB inhibition is a well-established effect of MG132 treatment as it blocks IκBα degradation, reducing NF-κB nuclear localization and DNA binding. The same group went on to identify the compound parthenolide as selectively inducing apoptosis in leukemia stem cells as opposed to normal hematopoietic stem cells, through a mechanism of increased reactive oxygen species, p53 activation, and NF-κB inhibition [159]. A subsequent *in silico* screen for additional drugs with specificity towards AML stem cells identified two other compounds, celastrol and 4-hydroxy-2-nonenal, and once again they found NF-κB inhibition to be part of the mechanism of action [160]. Parthenolide has also shown preferential activity in breast CSCs compared to the bulk tumor cells [161]. Use of SN50, a peptide inhibitor that blocks nuclear import of NF-κB and other transcription factors, decreases the sphere formation ability and tumorigenic capacity of glioma CSCs [162]. Others have found that inhibition of NF-κB promotes more rapid differentiation and progression to senescence in glioblastoma CSCs [163].

The activated B-cell subset of diffuse large B-cell lymphoma (DLBCL) has shown a distinct dependence on NF-κB signaling. Standard treatment for lymphoma patients includes rituximab, a monoclonal antibody against CD20. While this drug has many effects, one aspect includes inhibition of NF-κB signaling to induce apoptosis [164]. More recently, ibrutinib, a BTK inhibitor, has been found to improve patient outcome in clinical trials. While this drug does not specifically target IKK, BTK represents a key intermediate between B cell receptor and NF-κB, and ibrutinib treatment decreases NF-κB signaling [165]. Previously discussed studies have found an impact of IKK/NF-κB inhibition on tumor growth. While these efforts didn’t specifically analyze CSC effects, if CSCs are primarily driving tumor initiation, we could interpret these results as having some effect on the CSC population. Direct IKKβ inhibitors showed efficacy in a mutant KRas, p53-null model of lung cancer [166,167]. In addition to inhibitors targeting the kinase activity, the NF-κB pathway can be inhibited by peptides encompassing the NEMO-binding domain (NBD) that block association of the IKK catalytic subunits with NEMO/IKKγ. Recently, use of an NBD peptide slowed tumor growth in both a human glioma xenograft and a genetic mouse model of glioma [168]. The NBD peptide has also shown efficacy in a canine model of DLBCL [169,170].

NF-κB signaling also has ties to mediating resistance to radiation and chemotherapy, so there could be utility in combining NF-κB inhibition with traditional cancer therapies. Early work found that expression of IκBα-SR sensitized cancer cells to ionizing radiation, daunorubicin, and CPT-11 (a topoisomerase inhibitor) [171,172,173]. More recently, use of NF-κB inhibitors in combination with temozolomide, adriamycin, or radiation has shown increased apoptosis in glioblastoma cells [174,175,176]. Doxorubicin-resistant glioblastoma stem cells upregulated expression of MDR1 through a PI3K-NF-κB pathway [177]. In another study, KRas-NF-κB signaling mediated resistance to EGFR inhibitors in CSCs [178]. Upregulation of IRAK1 drives NF-κB activation and cytokine production, leading to CSC enrichment and paclitaxel resistance in breast cancer [179]. Taken together, these results suggest that not only could NF-κB inhibition be an effective treatment against CSCs, but it could also restore sensitivity to other therapeutic options.

## 5. Conclusions

The NF-κB pathway is integrated into many critical aspects of tumor biology. Its function in inflammation and immune responses often sets the stage for tumor development. Expression of several potent oncoproteins, including mutant RAS and BCR-ABL, leads to NF-κB activation early in tumorigenesis. Here, we have detailed crucial roles and contributions of NF-κB in cancer stem cells, which are driving tumor initiation, recurrence, and metastasis. NF-κB regulation of critical target genes—prominently including IAPs, cytokines, and EMT transcription factors—drive CSC phenotypes. In addition to direct NF-κB effects, there is also cooperation between other crucial CSC-associated pathways, such as STAT3, Notch, and TGF-β. Future work will need to determine if therapeutic targeting of the NF-κB pathway impacts tumor growth at the level of the cancer stem cells.

## Abbreviations

The following abbreviations are used in this manuscript:

AML: acute myeloid leukemia
APC: adenomatous polyposis coli
CML: chronic myeloid leukemia
CSC: cancer stem cells
DEN: diethylnitrosamine
DLBCL: diffuse large B cell lymphoma
ECM: extracellular matrix
EGFR: epidermal growth factor receptor
EMT: epithelial-mesenchymal transition
HSC: hematopoietic stem cell
IκBα: nuclear factor of κ light polypeptide gene enhancer in B-Cells inhibitor, α
IKK: inhibitor of κB kinase
LPS: lipopolysaccharide
NBD: NEMO binding domain
NF-κB: nuclear factor of κ light polypeptide gene enhancer in B-Cells
NIK: NF-κB-inducing kinase
POSTN: periostin
RANK: receptor activator of NF-κB
ROS: reactive oxygen species
STAT: signal transducer and activator of transcription
TLR: toll-like receptor
